# Family and domestic violence policy discourses and narratives: implications for Emergency Departments and communities in rural Australia

**DOI:** 10.1186/s12939-023-01873-y

**Published:** 2023-04-10

**Authors:** Sheree Moore, Rachael Fox, Bróna Nic Giolla Easpaig, Linda Deravin

**Affiliations:** 1 School of Psychology, Charles Sturt University, Wagga Wagga, NSW Australia; 2 College of Nursing and Midwifery, Charles Darwin University, Casuarina Campus, Darwin, NT Australia; 3 School of Nursing, Paramedicine and Healthcare Sciences, Charles Sturt University, Panorama Avenue, Bathurst, NSW Australia

**Keywords:** Family, Domestic, Violence, Policy, Australia, Rural, Remote, Emergency, Hospital, Discourse

## Abstract

**Background:**

Australian data has indicated that the frequency and severity of family and domestic violence (FDV) tends to increase with remoteness. Rural communities rely on Emergency Departments (ED) within public hospitals for general health and safety needs. Public health departments within Australia are strongly influenced by Government policies which can define ‘health problems’ and limit institutional responses to patients presenting with FDV. The current study therefore aimed to critically examine FDV Australian Government policies to explore how policy meanings could potentially impact on ED staff and individuals within rural communities.

**Methods:**

Foucauldian Discourse Analysis and Policy Narrative Analysis were used to examine 9 policy documents which represented national, state/territory and clinical practice levels. Publication dates ranged from 2006 to 2020.

**Results:**

A total of 8 discourses were identified, with each one providing a unique construction of the target problem and determining the potential agency of health professionals and subjects of FDV. Discourses combined to produce an overall narrative within each policy document. Narrative constructions of the target problem were compared which produced three narrative themes: 1) Deficit Subject Narratives; 2) Object Oriented Narratives; and 3) Societal Narratives.

**Conclusion:**

The results reflected a transition in the meaning of FDV within Australian society and over the past decade, with policies trending away from Deficit Subject Narratives and towards Object Oriented or Societal Narratives. Institutional systems, sociohistorical context and broader societal movements may have shaped this transition by stagnating policy meanings or introducing new insights that expanded the possibilities of understanding and action. Narratives produced assumptions which significantly altered the relevance and agency of individuals and groups when applied to a rural ED setting. As FDV was moved out of the clinical space and into the public domain, the agency of health professionals was reduced, while the values and strengths of FDV subjects and rural communities were potentially recognised. Later policies provided contextual specificity and meaning fluidity that could benefit diverse groups within rural areas; however, the expectation for ED staff to learn from their communities and challenge institutionalised approaches to FDV requires careful consideration in relation to rural hospital systems and resources.

## Background

In recent years, family and domestic violence (FDV) has become a national priority within Australia. Statistics have shown alarming rates of abuse in intimate relationships, including physical and sexual violence (1 in 6 women and 1 in 16 men) and emotional abuse (1 in 4 women and 1 in 6 men) [1]. Australian Governments, Non-Government Organisations (NGO’s) and the research community have responded to this concern, with agencies generating policies and publications which aim to reduce rates of FDV. In 2011 the Council of Australian Governments (COAG) released a FDV policy document commonly referred to as ‘The National Plan’ [2] which outlined recommendations for improving rates of FDV across the nation and over a 12 year period. National, state and territory Governments were expected to respond to the following six outcome areas: changing public attitudes related to FDV against women and children, fostering respectful relationships amongst young people, strengthening First Nations communities, improving services for women and children, improving the justice system response, and holding perpetrators to account for FDV. State and territory Governments have since released frameworks and strategies specifying responses within each jurisdiction; however, the impact of these documents is largely unknown and national data collected in 2016 suggested that rates of FDV had remained relatively stable over the preceding decade [1]. Rural areas appear to be particularly vulnerable, with data indicating that the frequency and severity of FDV increases with remoteness within Australia [3]. Frontline health services such as Emergency Departments (ED) may play a crucial role within these areas, with individuals in remote and very remote communities being 24 times more likely to be admitted to hospital as a consequence of FDV compared to those living in urban areas [1] and those who have been hospitalised for FDV being 10 times more likely to die from an assault [4]. Reflecting on FDV trends occurring throughout the nation, in 2022 the Commonwealth Government of Australia published a new national FDV plan for the period 2022 to 2032 [5]. This document acknowledged ongoing concerns, among which included service gaps in rural areas, ‘systems abuse’ which inadvertently encouraged FDV, and the inadequacies of mainstream institutions associated with criminal justice, housing and health (p. 55–71).

Public policy empowers Government to define ‘health problems’ [6], enabling them to control resource allocation and influence institutional systems, processes and individual behaviours [7]. Institutional discourses contained within policies can form explicit and implied understandings of the target problem and contribute to the agency of individuals or groups by positioning them in certain ways relative to others [8]. This contributes to assumptions of meaning and action by producing ‘policy narratives’ that depict who is responsible and who has the ability to act [8, 9]. Made up of discourses which can represent broader sociohistorical understandings, these narratives can be enacted and replicated by health professionals within public institutional settings [8]. The implementation of Government FDV policies can therefore create or reinforce uniform (and potentially problematic) ideas or actions inherent within these narratives, encouraging health professionals to make certain generalisations when responding to individuals who may present with FDV. Government understandings of FDV within public policy may inform the clinical practices of public health professionals in this manner, for example by influencing perceptions of whether a patient has a legitimate ‘health problem’ or the ability to help themselves.

Previous research investigating FDV discourses within Australian institutional documents has identified differing terminologies and meanings which may contribute to ongoing service response issues, with explanations of FDV remaining largely inconsistent within research, policy and legislation [10-12]. Some definitions are claimed to produce oversimplified meanings, potentially encouraging responses which overlook certain types of abuse or the impacts of broader factors [13]. For the purpose of the current study, the term ‘family and domestic violence’ (FDV) has been used to align with recent Australian Government departments and to acknowledge the complexity of FDV which can involve different types of abuse occurring within the context of intimate or family relationships [14]. The framing of FDV may also significantly change a policy’s focus, and shape institutional responses through the exclusion or targeting of certain groups. Dichotomous constructs may encourage hierarchical comparisons, creating rigid, generalised and contrasting assumptions which may contribute to inequality [15]. Some Australian policies have been criticised for reinforcing gender inequalities or stereotypes through masculine perspectives which encourage male privilege [13]. Murray and Powell [16] discuss how a gendered approach to FDV can benefit Australian women by encompassing broader disadvantages related to gender inequality; however, some binary gendered constructions can encourage ‘protectionist discourses’ which normalise problematic gendered assumptions (e.g. that women are vulnerable) and practices (e.g. excluding others from FDV interventions) [16, 17]. The context in which FDV is positioned may also alter meanings and actions. Kuskoff [13] warns against placing FDV within a public community setting, claiming that this may take the focus away from perpetrator responsibility and create problems when public citizens are intervening without sufficient understanding of FDV. The terminologies, framing and context of a policy can determine the possible assumptions and consequences for individuals and certain groups. For example, deficit constructions of First Nations people of Australia have been linked to paternalistic responses and homogenous stereotypes of deficiency and inferiority thought to be influenced by colonial ideology [18, 19], and this has been related to some health practices within rural settings [20].

International investigations into the experiences of rural communities have highlighted the importance of specificity and context when considering the effectiveness of policies and interventions aimed at reducing rates of FDV. A literature review of intimate-partner violence (IPV) in the USA indicated that while overall rates of IPV did not differ significantly between rural and urban locations, there were particular impacts within rural communities [21]. IPV tended to be more chronic and severe, outcomes for survivors worse, homicides more likely, and the prevalence was higher within certain rural groups. Canadian research has identified a number of barriers for rural communities impacted by IPV, including those related to geography, culture, service system gaps and the complexity of individual needs [22, 23]. A study investigating IPV in Sub-Saharan Africa also indicated that women in rural areas with lower educational levels were at greater risk [24]. These results may indicate the potential for intersectionality which places an individual at greater risk of FDV due to the cumulative impacts of multiple disadvantages, one of which may be geographical location [5, 25, 26]; however, there have been recent claims that current Australian FDV policies fail to recognise the importance of this for marginalised groups [27].

Despite evidence that Australian rural communities are experiencing higher rates of FDV [3], there is limited detailed information regarding FDV within these areas and rural communities are not homogenous. The term ‘rural’ is used in the current article to refer to a vast range of populations, cultures and geographies located within outer regional, remote and very remote locations as specified by the Accessibility and Remoteness Index of Australia (ARIA +) [28]. Nevertheless, information that does relate to Australian rural communities suggests similar challenges to those mentioned above, as well as many other factors such as stigma towards FDV and related health issues, limitations to privacy and confidentiality, social/economic disadvantages, systemic barriers and service gaps, and gendered attitudes which can encourage female oppression and abuse [3, 29-33]. Contextual details such as these can be vital to understanding why FDV appears to be different in rural locations. The increased rates of FDV in rural areas and the intersecting disadvantages of some rural communities reiterates the need for contextual specificity in policy and practice; however, Australian government policies have been criticised for taking a general approach which may overlook the importance of rural context [34].

Responses to FDV which are outlined within recent Australian Government policies vary between jurisdictions and a detailed explanation of every jurisdiction is beyond the reach of this article; however, responses can involve a range of private and public services, including police, health services, child protective services, housing support, services related to specific ‘at-risk’ groups and specialist FDV services. There are several reasons why rural ED’s may need to respond to FDV. Australian ED’s offer a ‘free’ Government funded 24 h service that can respond confidentially to a range of health and wellbeing concerns, potentially allowing residents to overcome certain barriers to accessing support [35]. FDV can impact on individuals at times or places where other services are not available, for example at night when services such as police and ED’s are still functioning. ED’s are not exclusive in their acceptance of referrals, being open to all Australian citizens regardless of gender or other characteristics. Furthermore, FDV has been linked to a number of health related concerns which commonly result in an ED presentation, including physical injuries, mental health conditions, pregnancy issues and chronic diseases [36, 37]. These factors can make ED’s a convenient or necessary alternative for support, and potentially the first point of contact for many survivors and perpetrators of abuse [5]; however, these departments are predominantly designed to respond to acute medical presentations and it is unclear if they are supported to respond effectively to FDV [35]. Australian and international research which have examined ED responses to FDV highlight the potential for inadequacies and challenges [38-42]. For example, ED health professionals can have limited resources for FDV, including time and specialist education [39, 42], and the emotional impacts of caring for survivors of abuse can impair their wellbeing and response to patients [43].

Australian ED’s are heavily tied to Government decisions, with public hospitals being funded and monitored by national, state and territory Governments [44]. Government policies and institutional processes within Australia have been previously criticised for their urban perspective, with claims that their relevance and application may encourage problematic understandings or responses when applied within marginalised settings [45, 46], such as assumptions which overlook the specific systems, needs or resources within rural communities [47]. For example, city hospitals often refer patients to Social Workers for concerns related to FDV [41]; however, specialists such as this may be absent or significantly limited in their availability within some rural hospitals, placing greater demand on ED nurses and doctors. There is also the potential for rural ED’s to be impacted by multiple policies due to the layered structure of Australian Government departments [48] and the tendency for institutional discourses to linger in spite of policy replacements [49]. Even policies which are not directly linked to FDV or to ED have the potential to impact on these departments and their rural communities; for example, by promoting practices which favour ED presentations other than FDV, or by moving FDV resources towards other services. The geographical location of a community and their access to relevant knowledge and services is also likely to play a role in determining whether ED’s can respond effectively to FDV or comply with Government policy suggestions. Rural ED health professionals may therefore be limited by public institutional discourses that impact on their department and local community; however, there is little detailed information regarding the functioning of rural ED’s, particularly in regards to FDV and how Government policy might impact on their responses.

### Study aims

The current study is linked to a larger body of work which investigates how Australian rural ED’s respond to FDV and why they might be responding in certain ways. The work attempts to provide an institutional and contextualised perspective by analysing data from three different sources: 1) policy documents, 2) focus groups with rural ED staff, and 3) observations within rural ED’s. This article outlines the results of the policy analysis which aimed to critically examine Australian Government FDV policies to explore: 1) how discourses construct policy narratives of FDV; 2) how this might influence a health professional’s understanding and response to patients presenting to ED with FDV; and 3) the potential implications for individuals within rural Australian communities.

## Methods

### Study design

Ethical approval for the study was granted by Charles Sturt University Human Research Ethics Committee (H20381; H21024). The study was designed to provide a national and historical perspective of Australian Government family and domestic violence (FDV) policies which were: 1) linked to public health institutions and 2) influenced by The National Plan [2]. In addition, the study aimed to provide geographical specificity by focusing on 3 state and territory jurisdictions: New South Wales (NSW), Western Australia (WA) and the Northern Territory (NT). The selection of these jurisdictions was influenced by a larger body of work which examines rural ED responses to FDV. The work highlights potential policy-to-practice disconnections and the challenges faced by staff when institutional policies and processes do not match up with individual needs and meanings of FDV within rural communities.

### Data collection

Policy documents were purposefully selected based on the above criteria. Government websites were used to identify relevant policies which were all publicly available. Policy documents were selected to provide contextual representation in respect to time (2006 to 2020) and jurisdictional ‘level’ (national, state/territory and clinical practice). The ‘clinical practice’ level involved documents which specifically targeted frontline public health responses. Table 1 below provides a list of 9 documents which were included in analysis. Each document will be referred to by its ‘data reference’ throughout the remainder of the article to ease readability. One document was dated prior to The National Plan [2] and it was included because it was the latest policy version associated with this jurisdictional level (i.e. NSW clinical practice). One document was jointly authored by non-government organisations (NGOs) and Victoria Health (i.e. Victorian state Government department for health) and it was selected due to its national perspective (i.e. level) and connection to the National Plan [2].

**Table 1:** Policy documents included in analysis

**Data Reference**^a^	**Year**^c^	**Jurisdiction**^b^	**Level**^d^	**Author & Title**
NSW06 [50]	2006	NSW	Clinical Practice	NSW Health; *Domestic violence: Identifying and responding;* p. iii
COAG11 [2]	2011	COAG	National	Council of Australian Governments (COAG); *The national plan to reduce violence against women and their children;* p. 2–3
NSW12 [51]	2012	NSW	State/ Territory	NSW Government; *It stops here: Standing together to end domestic and family violence in NSW: The NSW Government’s domestic and family violence framework for reform;* p. 2–3
WA14 [52]	2014	WA	Clinical Practice	Department of Health WA; *Guideline for responding to family and domestic violence; p. 3–4*
NGO15 [53]	2015	NGO & VIC	National	Our Watch, Australia’s National Research Organisation for Women’s Safety (ANROWS), and VicHealth; *Change the story: A shared framework for the primary prevention of violence against women and their children in Australia;* p. iii
NSW16 [54]	2016	NSW	State/ Territory	NSW Ministry of Health; *NSW domestic and family violence blueprint for reform 2016–2021: Safer lives for women, men and children;* p. 1
NT18 [55]	2018	NT	State/ Territory	NT Government; *Domestic, family and sexual violence reduction framework 2018–2028;* p. 5
NT20 [56]	2020	NT	Clinical Practice	NT Government; *NT domestic and family violence risk assessment and management framework;* p. 7–9
WA20 [57]	2020	WA	State/ Territory	Department of Communities; *Path to Safety: Western Australia’s strategy to reduce family and domestic violence 2020–2030;* p. 10–11

^a^Data Reference: Reference used within the text to indicate the policy document

^b^Jurisdiction: The document’s relevance when considering author responsibilities related to national or state/territory Governments. *COAG* Council of Australian Governments, *NGO* Non-government organisation, *VIC* Victoria, *NSW* New South Wales, *NT* Northern Territory, *WA* Western Australia

^c^Year: The document’s year of publication

^d^Level: The document’s intended audience or application level based on the following categories: *C* Clinical Practice, *S* State/Territory, *N* National

### Data analysis & methodology

Each document was initially examined to identify a summary section (1 to 3 pages in length) which was selected for analysis. Relevant pages for each section are indicated in Table 1. This was done for a number of reasons. Firstly, the narrative approach to analysis (described below) required that the text contained components of a ‘policy story’. This included characters (i.e. subjects), narrative elements (e.g. plot or structure), and the formation of meanings which constructed ‘problem’ and ‘solution’ streams [58, 59]. Second, policy stories needed to be compared between documents and the summary section was a consistent feature within every document. Third, examination of each document indicated that the summary section represented the dominant discourses that were repeated throughout the document and focusing on a specific piece of text allowed for an in-depth analysis of these discourses.

Foucauldian Discourse Analysis [8, 60] and a form of Narrative Policy Analysis [59] were used for each policy section. Foucauldian Discourse Analysis was initially used to critically examine distinct discursive formations which created unique ‘objects’ (i.e. the problem in need of change), ‘subjects’ (i.e. individuals, organisations or groups of people with assumed characteristics) and ‘contexts’ (i.e. conditional or spatial assumptions) linked to the construction of FDV [60, 61]. A discursive formation represents a separate ‘discourse’ that is defined by rules and which is differentiated by a threshold of conditions that support certain assumptions and possibilities [62]. This approach involved a detailed analysis of discursive conflicts and differences to distinguish boundaries in the transformation of meaning [62, 63] and to identify the potential for resistance [64]. Queer Theory aided in recognising categorical and dichotomous assumptions that could be problematic for certain groups [65].

Utilising Foucault’s understanding of institutional power, these discourses are not considered to be objective reflections of the ‘truth’, nor are they discursive tools that are purposefully selected by the authors; rather, they are considered to represent historical deviations in meaning that have been supported and reproduced within institutional systems and procedures [66]. The power of institutional discourses lies in their ability to limit what can be seen, said or done within a particular time and place [67]. Institutional discourses categorise, problematize, position and frame objects and subjects, thereby creating assumptions in respect to their qualities and agency [61, 68]. Discourses are therefore associated with power, as they have the potential to constrain, exclude, or produce in varying ways and within relevant or unexpected spaces [61]; however, a discourse can significantly differ in regards to its influence, with the potential to produce negative, neutral or positive effects depending on perspective and context, and the influence of other discourses [69, 70]. A symbolic approach is used within this analysis to demonstrate the productive potential of these discourses, an approach which is reflected in some of Foucault’s original work, such as the description of how “the whole dark underside of disease came to light, at the same time illuminating and eliminating itself like night, in the deep, visible, solid, enclosed, but accessible space of the human body” (p.195) [8]. Consistent with Foucauldian notions of ‘spaces’, this approach seeks to recognise the contextual conditions and abstract nature of discursive formations [61, 71].

Within a single policy document there can exist several discourses which are woven together to form an overarching narrative within a problem–solution policy structure [59]. While it is acknowledged that a single discourse is not used intentionally, literally or in isolation of all other possible meanings, the Foucauldian portion of the analysis aims to tease apart and isolate these discursive constructions of FDV to reveal them to the reader and allow for their underlying assumptions to be challenged [63, 66]. The Narrative Policy Analysis was influenced by Winkel and Leipold [59] and was used following Foucauldian Discourse Analysis to gain an understanding of how discourses worked together to produce a dominant meaning or ‘narrative’ for each document. The discourses within each document were classified based on the nature of their influence (i.e., dominant, supportive or counter) and their contribution to policy ‘streams’ (i.e., problem or solution streams). This is reflected in the tables attached to each narrative theme within the results section. Dominance was determined by the frequent or consistent use of the discourse within and across policy streams. Supportive discourses enhanced the meanings of dominant discourses within the text, creating a central theme within the narrative. Counter-discourses contrasted with the dominant theme, creating conflicts or complexities in meaning. The conflicting nature of counter discourses represents the potential for resistance towards dominant meanings and actions [64]. Policy narratives were compared and grouped into narrative themes which were then examined for potential sociohistorical influences. Emphasis was placed on meanings related to the construction of the target object (i.e. the problem in need of change) and how this potentially impacted on the agency of individual subjects and health professionals. Consistent with Foucauldian ideas of framing, the narrative portion of the analysis recognised how discourses influenced narrative tones, such as assumptions of tragedy or optimism [69].

The analysis maintained a focus on the context of a rural Emergency Department (ED) to understand the possibilities of policy translations ‘on the ground’ within these areas. Despite this frame of reference, the authors aimed to remain open to the possibility that FDV could be constructed as a ‘non-health’ or ‘non-rural’ object and to demonstrate how seemingly irrelevant constructions such as this could have an impact within a rural ED. This approach was used to understand how Government policy could potentially influence the ways in which health professionals within rural ED’s may understand and respond to individuals impacted by FDV.

## Results

As indicated in Table 1 above, the 9 policy documents involved in the analysis represented national (*n* = 2), state/territory (*n* = 4) and clinical practice levels (*n* = 3). NSW, WA and the NT each had at least one document at state/territory and clinical practice levels. Documents ranged in publication year from 2006 to 2020.

Table 2 below summarises the results of Foucauldian Discourse Analysis and Policy Narrative Analysis. The table provides a brief description of each discourse and narrative based on object meaning and subject position, and identifies the documents pertaining to each one. Documents are arranged in a manner that allows for sociohistorical comparisons regarding publication date, jurisdiction and level.

**Table 2:** Summary of results for Foucauldian Discourse Analysis and Policy Narrative Analysis

Discourses & Narratives			Policy Documents									
			NSW [50]	COAG [2]	NSW [51]	WA [52]	NGO & VIC [53]	NSW [54]	NT [55]	NT [56]	WA [57]	^a^** Jurisdiction**
			2006	2011	2012	2014	2015	2016	2018	2020	2020	^b^**Year**
			C	N	S	C	N	S	S	C	S	^c^**Level**
**Discourses**	**Objects**	**Subjects**										
*Biomedical Deficit*	FDV as a pathogen within an individual	Passive patient	X	X	X	X			X			
*Deficit Female*	FDV as a binary gendered object FDV as a ‘woman’s issue’	Disabled women Abled men	X	X								
*Protecting Children*	FDV as an intergenerational cycle of harm and criminality FDV as a symptom of poor parenting	Child victim Bad/Incapable parents							X			
*Criminalising*	FDV as a ‘bad’ action attached to time and place	Victim Villain		X	X	X		X	X	X	X	
*Social Justice*	Inequality within society (which causes FDV)	Society Disadvantaged group			X		X	X	X	X	X	
*Public Epidemic*	FDV as an infectious disease FDV as a societal epidemic	Societal patient				X	X		X		X	
*Evolutionary*	FDV as an evolving historical object detached from individuals	Societal objects of research					X				X	
*Feminine Oppression*	Binary gendered inequality within society (which causes FDV) Feminine disadvantage within society	Disadvantaged feminine group					X					
**Narratives**	**Objects**	**Subjects**										
*Deficit Subject*	FDV as a private object attached to the individual FDV as a stereotypical object	Individuals as incapable or defective Professionals as experts & heroes	X	X	X	X			X			
*Object Oriented*	FDV as an object detached from individuals FDV attached to time and place	Individuals with potential for strength and change Individuals with position versatility Professionals as experts & heroes						X		X		
*Societal*	Social inequality as the target object (which causes FDV) FDV as an object located within society	Societal patient Society as a social responder Communities as leaders & experts					X				X	

^a^ Jurisdiction: The document’s relevance when considering author responsibilities related to national or state/territory Governments. *COAG* Council of Australian Governments, *NGO* Non-governmant organisation, *VIC* Victoria, *NSW* New South Wales, *NT* Northern Territory, *WA* Western Australia

^b^Year: The year the document was published

^c^Level: The document’s intended audience or application level based on the following categories: *C* Clinical Practice, *S* State/Territory, *N* National

The results of *Foucauldian Discourse Analysis* are discussed in detail below Table 2. This is followed by the results of *Policy Narrative Analysis* which can be found under the corresponding heading. Sociohistorical comparisons are further addressed within the Discussion section.

### Foucauldian discourse analysis

Each document was initially analysed using Foucauldian Discourse Analysis to identify discourses which contributed to: 1) the construction of a target problem in need of change; and/or 2) the position of individual subjects and health professions. There were 8 discourses in total which are summarised in Table 2. The number of discourses within a single policy document ranged from 2 to 5.

**Fig. 1:**
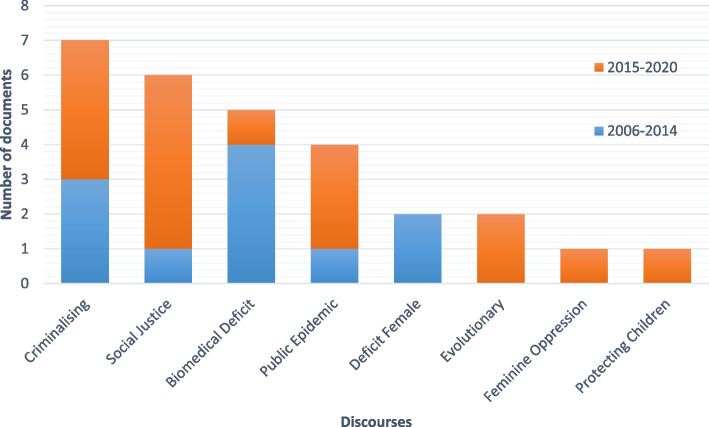
Policy Discourses: Number of documents and year of publication

Figure 1 below reflects the number of documents which related to each discourse. To provide a historical perspective, documents have been categorised into publication year-groups: 1) 2006–2014 and 2) 2015–2020. Biomedical Deficit, Public Epidemic, Criminalising and Social Justice were the most common discourses found within policy documents. While Criminalising discourse had a relatively consistent influence over time, the influence of other discourses changed between year groups, with Biomedical Deficit and Deficit Female dominating earlier policies, and Social Justice and Public Epidemic favouring later documents. Later documents also appeared to be influenced by new discourses such as Evolutionary, Protecting Children and Feminine Oppression discourses, creating the potential for greater variability in meaning. Protecting Children and Feminine Oppression were found exclusively within particular documents, making NT18 [55] and NGO15 [53] stand out within the sociohistorical trends of other documents and this is addressed in more detail within the Discussion section. As indicated in Table 2, object meanings and subject positions differed significantly between discourses. Each discourse is described in detail below.

### Biomedical deficit discourse

Biomedical Deficit Discourse constructs family and domestic violence (FDV) as a pathogenic object attached to the body and mind of an individual, causing symptoms that cripple their independence and strength.

*‘Domestic, family and sexual violence is a serious, prevalent and life-threatening problem…[with] profound physical, psychological, social and economic effects …[including] serious injury, disability or death, chronic pain and disease, mental health issues, loss of employment, absenteeism and presenteeism, financial insecurity and isolation, and alienation from family and social support.’* (NT18 [55], p. 5).

There is a succession of deficits related to health, finances and employment, and a passivity brought about by suggestions of absence and social disconnect. Longevity or permanency is implied through suggestions of chronicity, disability, and death. Assumed to be connected to a single subject, these outcomes construct a patient subject who is starkly abnormal, being laden with physical, psychological, economic, vocational and social inabilities. The possibility of strengths and abilities is lost as they are consumed by the sickness of FDV and assumed to be unable to help themselves.

In contrast to the pathologised patient subject is an image of a strong and resilient figure who is able to have *‘control and have power over another person in an ‘intimate’ or family relationship’* (WA14 [52], p. 3). The dominance of this subject allows them to escape the symptoms of FDV, while the patient subject takes centre stage as the problem target in need of treatment and change. Consequently, actions turn away from the ‘perpetrator’ of FDV and are focused on the ‘victim survivor’, requiring others to put *‘the victim at the centre of the system so they can get the services and support they need’* (NSW12 [51], p. iii), such as *‘ongoing treatment and follow-up counselling’* (NSW06 [50], p. iii).

The patient subject is therefore dependent upon the judgements, actions and inactions of others; while the dominant subject is positioned as an object on the periphery of this construction, rendering them redundant within the solution. A deficit and pathologising lens disempowers the victim survivor, allowing for them to be ‘rescued’ by heroic professionals who are constructed as experts needed to identify and respond to the pathogenic disease. Consequently, Biomedical Deficit Discourse makes the victim survivor the target problem in need of change. Their position of dependence assumes that health professionals will take control over the patient’s body and mind. While the discourse may elicit feelings of sympathy towards the patient subject due to an emphasis on their suffering, the presumption of inability can provide a patronising rather than compassionate stance and encourage health professionals to overlook the potential for strength.

The construction of FDV as an object attached to the body and the controlling nature of this discourse mimics some of Foucault’s description of the medical industry [8]. Biomedical discourses have been previously mentioned and criticised for their disempowering, deficit and pathologising focus on patients and certain groups due to dichotomies such as normal-abnormal [72-75], with evidence of these constructions producing problematic health practices [76]; however, some biomedical discourses may offer clarity and link patients to necessary treatments, hinting at the possibility for benefits in certain situations [77].

### Public epidemic discourse

Public Epidemic Discourse places emphasis on the social and societal development of FDV which is constructed as an infectious disease that threatens society. FDV is positioned within the public domain, contaminating the *‘social, political and economic structures, practices and systems’* (NGO15 [53], p. iii) that surround the individual. Its social quality enables it to spread within society with the suggestion that it moves beyond an initial subject to impact on ‘*children who are exposed to it, their extended families, their friends, their work colleagues and ultimately the broader community’* (COAG11 [2], p. 2). The implied infectiousness of FDV creates a catastrophe akin to an epidemic, demanding solutions focused on *‘individual, family and whole-of-population levels’* (WA20 [57], p. 11).

Society takes up the position of patient, while services are given responsibility and are criticised for their imperfections, since *‘while some excellent services are already in place, often the right service is not available at the right time to meet needs’* (WA20 [57], p. 11). The value of current services is undermined by their inadequacies as they appear to precipitate FDV by allowing society to fall into gaps where individuals succumb to the disease. This allows Government to take up a parental position where they are able to *‘reform systems to prioritise safety, accountability and collaboration’* (WA20 [57], p.11), leading to assumptions that society will be ‘rescued’ from services which are currently ‘unsafe’ or are somehow failing to take responsibility for FDV. Implied disconnections construct services as siloed, leading to solutions which attempt to cover these potential gaps.

*‘Government agencies and community sector services will work together to provide culturally appropriate, holistic and accountable responses to victims and perpetrators, streamlined pathways through the service system, and coordinated service delivery between agencies and systems…across the continuum of primary prevention, early intervention and crisis response.’* (WA20 [57], p. 11)

Words such as ‘streamlined’, ‘holistic’ and ‘continuum’ give a sense of saturation that provides certainty for control. The solution appears to counteract the infectious spread of FDV by creating an apparently flawless flow of service support which is assumed to reach everyone within the author’s jurisdiction. This confident stance is supported through the description of FDV as *‘a product of complex yet modifiable social and environmental factors’* (NGO15 [53], p. iii), which simultaneously validates the challenges of FDV while providing an optimistic expectation of control.

Public Epidemic Discourse identifies services as the target problem in need of change, providing a critical stance which undermines their current agency and value. Despite this, some acknowledgement of ability and strength encourages assumptions that go beyond suggestions of blame and towards improvement. Recognition of broad societal factors removes FDV from ‘the individual’, bringing about the possibility for prevention or early intervention which may be beyond the reach of current services, and this may offer validation and hope for health professionals. The individual subject is distanced from the issue as external factors and services are presumed to have responsibility in their stead; however, their implication in the spread of FDV assumes that they may become passive objects targeted for treatment. The discourse’s broad perspective and generalising potential may encourage problematic responses from health professionals who could overlook the specific needs and differences of individuals, as universal solutions aim to achieve change ‘for the greater good’.

Public Epidemic Discourse appears to contain elements of biomedical discourses. Foucault [8] discussed the spread of the ‘medical gaze’ into the social space, pointing out contradictory assumptions which aimed to nationalise medicine through the “strict, militant, dogmatic medicalisation of society”, while reducing the relevance of medicalisation through the eradication of disease via “a corrected, organised, and ceaselessly supervised environment” (p. 32). This description reflects the controlling and ambitious nature of this discourse. Despite these origins, Public Epidemic Discourse appears to break through the thresholds of traditional biomedical discourses by re-framing and contextualising the ‘disease’ in a manner which pushes it beyond ‘the individual’ and ‘the private clinic’, allowing for influences and actions that extend past the ‘body and mind’. This discourse appears to replicate constructions often found within an approach referred to as ‘Public Health’ [78, 79] which has historical ties to the World Health Organisation and which focuses on improving the health of populations or groups by drawing upon knowledge from medical, social, political, economic and environmental sciences. This discourse may therefore have the potential to embrace knowledges other than ‘biomedical’ and contribute to outcomes other than ‘medical domination’, such as the empowerment of marginalised groups. Public Health literature has previously described FDV as infectious and compared it to epidemics such as COVID-19, with a list of ‘risks’ and ‘impacts’ appearing to justify the urgent need for action on a societal level [80, 81].

### Criminalising discourse

Criminalising Discourse constructs FDV as a criminal act, emphasising who and what is ‘bad’. References to *‘assaults’* (p. 2) and *‘acts of violence that occur between people’* (COAG11 [2], p. 3) construct FDV as a singular physical event attached to a particular time and place akin to a crime scene. These acts of violence are *‘unacceptable’* (NSW06 [50], p. iii) and *‘inexcusable’* (NT20 [56], p. 7), with descriptions of ‘bad’ behaviour *‘which is violent, threatening, coercive or controlling, causing … fear’* (NSW16 [54], p. 1). These bad behaviours appear to accumulate and are attached to a single subject who is given full responsibility for FDV.

*The person responsible for FDV is the only person to be held accountable…and there is no rationale acceptable as an excuse to minimise the intent, extent or degree of harm caused by the person responsible.* (WA14 [52], p. 4).

With responsibility being piled onto a single subject, FDV can be seen to originate from within the individual who is the epitome of bad, constructing a unidimensional ‘villain’ subject who is positioned as a societal threat. There is little possibility for leniency or forgiveness, as the villain’s innate badness gives no impression for change. The villain therefore becomes the target problem with solutions focused on punitive action, as *‘[FDV] will not be eliminated unless perpetrators are held to account…via the justice system’* (NSW12 [51], p.3).

In contrast to the dominant nature of the villain is a defenceless victim who is crippled by the experiences of *‘physical, sexual and/or psychological damage, forced social isolation, economic deprivation, or…fear’* (WA14 [52], p. 3). Suggestions of secrecy and shame enhance the victim’s vulnerability, as FDV is seen to be hidden from public view.

*‘Violence within families is often hidden [and] women who are abused are frequently treated within NSW Health services, however they generally do not present with obvious trauma, even in Emergency Departments.’* (NSW06 [50], p. iii)

Portrayed as muted, the victim takes on potential qualities of deception or ignorance, as they presumably withhold information from others outside the home. Their cowering and crippled nature enables their silence and allows them to become potential accomplices to the perpetuation of FDV. Being present within the home, children are positioned as innocent witnesses, holding information of value which could be used to implicate their parents in the act of FDV.

The criminal nature of FDV positions health professionals as detectives, as they search for evidence attached to individuals who can be *‘identified or suspected of experiencing family and domestic violence’* (WA14 [52], p. 3). The badness of the villain and the muted nature of the victim makes them enemies unto themselves and others, justifying the privileged actions of professionals via *‘legal, policy and practice approaches’* (p.8), such as sharing subject information *‘without consent in certain situations’* (NT20 [56], p. 8).

The discourse focuses attention on the ‘villain’, making the perpetrator the primary target in need of change; however, the deficit, non-forgiving and suspicious stance encourages assumptions which implicates others within home. Individual subjects are therefore disempowered within this discourse, while professionals hover above them as authoritarian parental figures, with special permissions to access, share and use personal information. Consequently, human rights can be pushed aside to prioritise the minimisation of ‘risk’, potentially allowing departments and professionals to avoid criticism in their decision-making and actions. Given the innate badness of the villain and the hidden nature of the object, there is little hope for prevention or cure and a reliance on reactive punitive solutions.

International research into ED responses has identified problematic practices which may reflect a Criminalising Discourse, such as the mistrust of patients and a reliance on stereotypical subject information (e.g. the timid demeanour of a patient) [82]. Research into Australia’s legal and criminal justice system has linked the criminalisation of FDV to problematic practices which can disempower victim-survivors [83] and similar concerns have been raised within literature concerning USA criminal justice systems [84-87]. Historical perspectives have also revealed the potential for criminal justice policies to legitimise systems, processes and practices of control over ‘social problems’ [49] and oversimplify responses by punishing acts of violence without addressing broader contextual factors [88].

### Social justice discourse

Social Justice Discourse focuses on the object of equality, with FDV positioned as an outcome of inequality within society. Equality is constructed through a ‘vision’ which represents the one and only pathway for a positive future, offering an enticing image of widespread benefit as Australia becomes *‘an exciting place to work and live as we all pull in the same direction towards a shared goal of safety, equality and respect for all’* (NGO15 [53], p. iii). The object of ‘equality’ is set as a future destination, allowing for impressions of current and past ‘inequality’ which become ‘*the core of the problem and…the heart of the solution’* (NGO15 [53], p. iii).

A disadvantaged subject group is separated out from the rest of society, becoming the target beneficiary for change.

*[Aboriginal Family Safety] is our first priority, in recognition of the disproportionate impact of family violence on Aboriginal women, children, families and communities and the need to respond to the different drivers of violence experienced by Aboriginal people (WA20 [*57*], p. 11)*

The disadvantaged subject is seen to be held down by societal factors, making them a victim of their external surroundings. Consequently, the object moves beyond ‘the individual’, as ‘inequality’ is seen to have independent power to ‘*contribute to social and cultural environments where violence occurs’* (NT18 [55], p. 5). Broader society is therefore implicated in the formation of the problem, making ‘everyone’ responsible for change.

An authoritarian leadership position uses inclusive language to provide a sense of togetherness, with claims that *‘we have listened’* (WA20 [57], p. 11), *‘our efforts are focused’* (WA20 [57], p. 11) and *‘we will commit’* (NT18 [55], p. 5). Expectations of loyalty and dedication, and constructions of inequality as a societal threat, justify a universal ‘call to arms’ approach and coax individuals toward the chosen path. Togetherness becomes essential within the solution where everyone is expected to *‘speak the same language, use the same tools, have the same understanding, and work together’* (NT20 [56], p.7). Togetherness is emphasised to such a degree that it paradoxically weakens individuals and diverse groups by reducing the importance of difference within society. Equality is therefore assumed to be achieved via homogeny, creating a ‘follower’ position whereby everyone is expected to comply with the vision. In contrast, a collective leadership position recognises the importance of diversity and allows for the empowerment of communities with actions being *‘tailored to the specific needs of the community’* where *‘local ownership and leadership…[is] of paramount importance’* (NSW12 [51], p. 3). This position makes possible multiple avenues for change and recognises the potential for strengths within typically marginalised groups, who may be positioned as ‘collaborators’.

Conflicting images of inequality and equality are set against a historical backdrop to propel the reader towards the author’s hopeful vision which follows a ‘negative past to positive future’ trajectory. Current societal processes and systems of inequality therefore become the target problem in need of change. Prevention, early intervention and cure are made possible by the discourses’ causal construction, whereby inequality leads to FDV; however, this may support assumptions that privileged individuals are immune to FDV, encouraging professionals to ‘look for’ FDV within certain ‘at-risk’ groups. Subjects and professionals are brought together within a societal group, with the presumption that responsibility is dispersed equally amongst them. The generalising nature of the discourse also has the potential to reduce the relevance and motivation of health professionals by moving FDV out of the ‘health’ setting. While the collective leadership stance recognises the importance of difference which may allow for decision-making to be shared amongst diverse groups, the focus on equality and the societal context provides the impression that individual subjects may still be overlooked to some degree.

Emphasis on freedom and equality within Social Justice Discourse highlights the influence of human rights movements which have been used to frame FDV [87]. The collective leadership position emphasises diversity, complexity and the impacts of multiple disadvantages, resembling elements of ‘intersectionality’ which has been linked to FDV [89, 90]. Previous research on FDV policy and practice within Australia has revealed constructions which appear to resemble the authoritarian leadership position of Social Justice Discourse. For example, Maturi and Munro [27] demonstrate how an institutional focus on supporting a particular disadvantaged group can paradoxically “erase difference” (p.12) and reproduce racial inequalities by reinforcing stereotypical assumptions, dominant ‘white’ ideologies and an ‘us and them’ dichotomy between groups. The conflicting authoritarian and collective leadership positions formed within Social Justice Discourse potentially demonstrates the influences of contradictory global trends such as decolonisation and the governing of marginalised groups [91].

### Evolutionary discourse

Evolutionary discourse creates a temporal backdrop which supports the impression of gradual change in object meaning. The object of FDV is seen to emerge from the past, providing a sense of discovery as the object is found and brought forth into public view.

*‘In recent years, through increased public awareness, Australians no longer consider violence against women and their children to be a private issue. No longer are we willing to accept the untold damage…’* (NGO15 [53], p. iii)

FDV is transformed by society’s awareness and perspective, as it is seen to morph from privately acceptable to publicly unacceptable. There is an impression of growth as the object is assumed to expand in terms of space and severity, becoming more noticeable with damage that can no longer be ignored. The object is therefore seen to evolve over time, being influenced by its external environment through *‘certain factors that consistently predict – or drive- higher levels of violence’* (NGO15 [53], p. iii). Subsequently, individuals are absolved of responsibility for the creation of the object which emerged ‘somewhere else’.

This construction provides an evolutionist position where the object can be observed through an objective lens, with claims that there is *‘much to contribute’* and *‘much to learn’* (NGO15 [53], p. iii). With this growing understanding of the object, there is an expectation of gradual control as Government plans to *‘grow primary prevention as a key pillar in our long-term commitment to address family and domestic violence’* (WA20 [57], p. 11). Consequently, there is an expectation that not all is known and that professionals may become responsible for future change when external factors can be identified and altered.

Evolutionary Discourse provides a historical perspective which detaches the object of FDV from ‘the individual’, making FDV the target problem without attributing responsibly or blame to subjects. Despite this, the tendency to overlook the importance of individuals decreases subject visibility, potentially allowing for survivors to remain unheard and perpetrators to escape accountability. As evolutionary observers, Government takes responsibility as the expert, ‘making sense’ of what has happened and predicting what might occur. Their superior knowledge gives them agency, while acknowledgement of the need for learning humbles their position by avoiding the assumption that all is known. Professionals may be held in limbo due to the discourse’s uncertainty and stage of ‘transition’ from unknown to known, positioning them as trusting students who wait for clarification and solutions from the Government. This discourse may therefore reduce the relevance of health professionals by removing the object from the clinical setting and making FDV seem like a non-health issue.

Prior research has acknowledged the historical emergence of FDV on the international agenda [92]. The evolving nature of FDV has been discussed in terms of historical changes to social concepts of ‘family’ and gendered norms [93], and women’s rights movements, including some Feminist influences of the 1970s and more recently the Me Too Movement [94, 95].

### Deficit female discourse

Deficit Female Discourse constructs FDV as a binary gendered object, placing ‘male’ and ‘female’ subjects within a private relational setting. A female subject is made up of *‘Australian women and girls’* (COAG11 [2], p. 2), with repetitive references to ‘women’ increasing their visibility and vulnerability. The female subject is linked to a string of devastation, being considered *‘at risk’*, *‘abused’* (NSW06 [50], p. iii), and experiencing *‘harm and suffering’* (COAG11 [2], p.3). A collection of negative descriptors builds a tragic image of despair and helplessness, which places the female subject in a position of complete oppression. Impressions of meekness are encouraged through the assumption that they are unable to escape their fate, as *‘the majority of people who experience this kind of violence are women – in the home, at the hands of men they know’* (p. 2) who *‘exercise power and control over women and their children’* (COAG11 [2], p. 3). Binary gendered positions are set within the home, conjuring up heterosexual impressions. Women take up the position of parent, with children being attached as objects of ownership, while the parental position of men is typically not made explicit. Children are therefore powerless within this construction of FDV, being tied to their mother’s fate of tragic suffering.

While the female subject is consistently constructed without strength or ability, the male subject can be absent, flexible or ambiguous. In addition to being potential perpetrators of FDV, *‘men are victims of domestic and sexual assault’* as well as *‘victims of violence from strangers and in public’* (COAG11 [2], p. 2). A sense of versatility is encouraged through their ambiguity, as the male subject lacks visibility and is barely mentioned. The discourse therefore allows men to escape with the possibility of innocence and strength, with the potential for them to avoid feelings of guilt or shame as they side-step unfavourable labels such as ‘perpetrator’ or ‘victim’.

Female survivors of FDV become the target in need of change. The enhanced visibility of the female subject leads to their disempowerment, with the discourse’s tragic tones allowing for deficit assumptions. Responsibility and blame rests on the shoulders of individual women, as it is expected that they will inevitably fail to protect themselves and the children attached to them. By conforming to heterosexuality, the discourse excludes those who identify as LGBTQIA + or women who could be considered as perpetrators of abuse. The discourse can foster sympathy towards women and children, creating the position of ‘hero’ or ‘rescuer’ for health professionals whose dominance is justified by the female subject’s inherent weaknesses and dire situation. The individualised construction supports solutions focused on the female body and mind, increasing the responsibility of professionals involved in women’s health, psychology or the welfare of children.

Previous literature has warned against over-simplified gendered constructions of FDV which can assume rigid stereotypes of the ‘male perpetrator’ and ‘female victim’ [96], and justify institutional systems of control related to gender, sexuality and the female body and mind [97]. This discourse replicates concepts related to ‘protectionist discourses’ which have been previously mentioned [16]. It encourages many of the assumptions that are outlined by Lapierre [98], who highlights the presumption of failed mothering in the deficit construction of women in FDV publications. The concept of ‘absent presence’ related to a binary gendered stereotype of FDV has also been previously discussed, with some claiming that the visibility of abused women and the contrasting absence of men within policy and practice can problematize women and construct the deficit female subject [99, 100]. Research has also revealed problematic assumptions and practices by health professionals who appear to be influenced by a gendered stereotype of heterosexual FDV constructions [101].

### Feminine oppression discourse

Feminine Oppression discourse constructs FDV as an outcome of female disadvantage within society, focusing on the object of ‘gender inequality’. The object becomes a societal issue, as it is set within the public domain and is formed by social processes, such as:

*‘…beliefs and behaviours reflecting disrespect for women, low support for gender equality and adherence to rigid or stereotypical gender roles, relations and identities’* (NGO15 [53], p. iii).

These social processes are attached to a societal subject which encapsulates everyone without exception and regardless of gender or other characteristics. They are accused of creating gender inequality via active or passive means of female gendered oppression, positioning society as the perpetrator within this construction.

Binary gendered assumptions are formed through references to ‘women’, allowing for male–female comparisons on a societal scale and constructing a disadvantaged feminine subject group.

*‘Violence against women has been shown to be significantly and consistently lower in countries where women’s economic, social and political rights are better protected, and where power and resources are more equality distributed between men and women’* (NGO15 [53], p. iii).

The feminine subject represents a homogenous group of ‘women’ presumed to have shared and equal disadvantage within a particular environment, with external factors determining their agency and value. A causal relationship is established, with the suggestion that gender inequality within societal systems leads to FDV. Male privilege or patriarchal power structures are implied, rather than made explicit, leaving behind a mere silhouette of the male subject who is disempowered by the discourses’ feminine emphasis.

Feminine Oppression Discourse positions women as innocent survivors of society’s wrongdoing, as they are held back by processes and systems beyond their reach. The oppression of women therefore becomes the problem and society a target for change. The causal link to FDV encourages assumptions that only women are adversely impacted by FDV and that all FDV is a result of gender inequality. Consequently, the discourse potentially excludes certain groups, meanings or actions, such as considering that non-feminine gendered individuals could be ‘disadvantaged’ or that women could take up oppressive positions. The public context and movement beyond ‘the individual’ has the tendency to decrease the relevance of health professionals, with solutions expected to be focused on broader social and societal processes.

Some Feminist theories of FDV have provided similar constructions, emphasising female disadvantage through patriarchal assumptions of domination [78]. There is argument that a feminine gendered frame of FDV is necessary to avoid the danger of overlooking gender inequalities if policies move towards a non-gendered construction [102]; however, an over-emphasis on ‘female oppression’ may distract from the significance of other factors or groups involved in this issue [103]. Gilson [104] defines a ‘reductively negative’ form of female vulnerability within some Feminist constructions of FDV which appears to represent concepts within the current discourse, such as the homogenising of the female group which is given an inferior status and the superimposing of feminine female gender with societal disadvantage and suffering (p.74–75).

### Protecting children discourse

Protecting Children Discourse constructs FDV as an object of child risk of harm, as children are seen to be abused, neglected and contaminated by their parents.

*‘Exposure to domestic and family violence also increases the risk of a child or young person experiencing other forms of abuse or neglect. We know that for children, exposure to domestic and family violence is highly correlated with child protection reports and may lead to cycles of youth offending.’* (NT18 [55], p. 5).

FDV becomes a potential form of abuse linked to other types of abuse, building an environment where children are unsafe and not cared for. Children seem to be contained within the harmful environments created by their parents, with their innocence being consumed as they are seen to move from victim to perpetrator positions. A cyclic and generational construction of FDV is formed, allowing for the infinite perpetuation of FDV where current FDV (in childhood) leads to future FDV (in adulthood), creating a ‘chicken and egg’ conundrum which renders the subjects as helpless.

With adults failing to protect their children from harm, FDV becomes a symptom of poor parenting. A deficit lens assumes that children and parents are unable to help themselves or each other as they are stuck in this intergenerational cycle of crime and suffering. This produces the need for external control which aims to achieve a future where:

*‘…children are safe, respected and free, and where young people can exercise consent and engage in respectful and health relationships throughout their lifetimes.’* (NT18 [55], p.5)

Children are brought to the fore as parents appear absent within this outcome, making chances of their forgiveness or recovery seem unlikely. Consequently, the safety, respect and freedom of children is assumed to be achieved through means of parental restriction or replacement. Government and professionals are able to take the place of parents, monitoring and controlling families with the strength of *‘policies and reform initiatives, especially those targeting children and young people’* (NT18 [55], p.5). Their level of control is assumed to over-ride the rights of families or individuals who are constructed as impaired.

Protecting Children Discourse creates a scene in which children and adults of FDV are potentially held within a cyclic fate of devastation and criminality; with solutions offering children the possibility of change. While parents are identified as the problem, they are assumed to have limited or no capacity for improvement, therefore allowing their replacement or restriction. The heroic and disciplinary positions of health professionals are justified by the need to ‘save’ children from their parents and the cycle of FDV. Emphasis on child risk of harm and its link to FDV may encourage professionals to overlook the potential strengths of parents and families, and the risks associated with family interventions. The threat of family separation or manipulation has the potential to elicit elements of fear or mistrust towards health professionals, as the risk of child harm takes precedence and the strengths, rights and needs of parents can be sidelined.

Previous literature mentions the intergenerational cycle of FDV which is thought to be associated with social learning theory [78]. Previous research examining discourses within child protective services have revealed similar deficit constructions, such as a diseased model of child abuse and forensic influences which create the potential for stereotypical assumptions and problematic practices driven by suspicion [105, 106]. Jack [105] discusses the threatening nature of such discourses which encourage a focus on gaining immediate control over families through compulsory actions and the standardisation of parenting, as opposed to keeping children safe within their homes.

## Narrative policy analysis

**Fig. 2:**
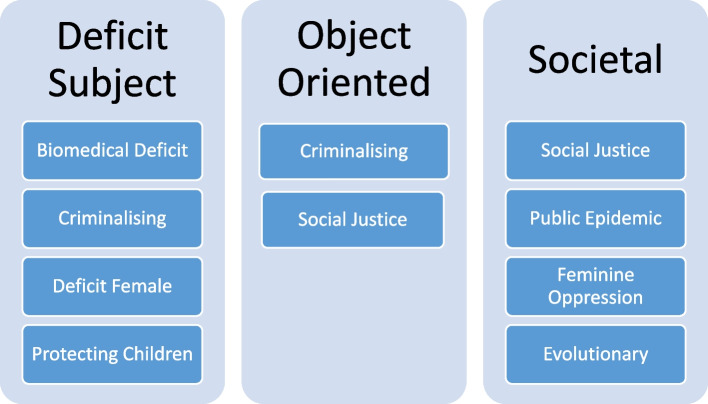
Discourses influencing narrative themes

Narrative policy analysis was used to examine how the unique combination of discourses within each document produced an overall storyline (or narrative) and the potential ramifications of this for health professionals and the subjects of FDV. The narratives demonstrate the potential for discourses to have varying effects depending on their interactions with other discourses. The analysis produced 3 narrative themes: Deficit Subject Narratives, Object Oriented Narratives and Societal Narratives. The results illustrate how policy narratives considerably changed the possibilities of meaning and action for individuals and groups by determining who or what the problem was (i.e. ‘objects’ constructed within the problem stream), what could be done about it (i.e. actions or outcomes constructed within the solution stream), and who had responsibility to decide, initiate or take action (i.e. subject positions). Table 2 above summarises the defining objects and subjects of each narrative theme, and identifies which documents contributed to the narrative construction. Figure 2 below outlines the discourses which heavily contributed to each narrative theme and this is followed by detailed descriptions.

### Deficit subject narratives

Deficit Subject Narratives locate the problem within the individual, constructing subjects as diseased or defective and identifying them as the target problem. Table 3 below outlines the discourses within the policy streams of 5 documents which had a Deficit Subject Narrative. Biomedical Deficit Discourse influenced all 5 documents, encouraging a focus on the survivor of FDV who could be considered as a diseased and crippled patient in need of rescuing. In 4 documents, the combined dominance of Biomedical Deficit and Criminalising Discourses provided a deficit perspective of perpetrator and survivor, disempowering subjects through assumptions of innate inability or badness. Deficit Female and Protecting Children Discourses encouraged this deficit perspective and enhanced the focus on particular subjects (i.e. women and parents respectively). Protecting Children and Criminalising Discourses contributed to a focus on urgent punitive solutions directed at parents and perpetrators, while Biomedical Deficit and Deficit Female Discourses implied interventions aimed at the survivor’s body or mind. These discourses tended to encourage stereotypical constructions based on personal characteristics such as gender.

**Table 3:** Deficit Subject Narratives: Documents and discourses

Documents	Problem Discourses	Solution Discourses
NSW06 (Clinical Practice) [50]	**Biomedical Deficit** *Deficit Female*	**Biomedical Deficit**
COAG11 (National) [2]	**Criminalising** *Deficit Female*	**Biomedical Deficit** *Deficit Female*
NSW12 (State) [51]	**Biomedical Deficit** **Criminalising** *Social Justice*	**Criminalising** **Social Justice** *Biomedical Deficit*
WA14 (Clinical Practice) [52]	**Biomedical Deficit** *Criminalising* *Public Epidemic*	**Criminalising** *Biomedical Deficit*
NT18 (State) [55]	**Biomedical Deficit** **Protecting Children** *Criminalising*	**Public Epidemic** **Criminalising** *Social Justice* *Protecting Children*

Bold – dominant; *Italics* – non-dominant

Public Epidemic and Social Justice Discourses were counter-discourses within 3 documents. They provided a conflicting or complex perspective when combined with the above discourses due to their societal focus. Social Justice Discourse in particular countered some of the tragic and pessimistic tones of other discourses by providing a vision of togetherness or hope for the subjects involved. Given the unique combination of these discourses within each document, narratives wavered in their expectation for individual change. Documents without the influence of counter-discourses tended to be more pessimistic (e.g. NSW06 [50]), with greater focus on the problem subject and a reliance on reactive solutions leading to impressions of eternal criminality or suffering for certain subjects.

These narratives provided tones of tragedy and heroism. They assumed a power dynamic whereby subjects can be completely dominated by or dependent upon health professionals. Positions of hero, expert or parent enable health professionals to protect, save or discipline subjects who are assumed to be disabled and therefore not able to help themselves. Health professionals are therefore empowered with decision making and action, and responsible for change. These narratives have the potential to promote problematic assumptions or responses by encouraging health professionals to overlook potential strengths and abilities of subjects and to rely on individual characteristics when considering FDV.

### Object oriented narratives

These narratives constructed FDV as an object which could be separated out from the individual, making it the target problem. This allowed for individuals to have position versatility and the potential for ability or strength. Table 4 below outlines the discourses involved within 2 documents which both had Object Oriented Narratives. Social Justice and Criminalising Discourses were the only two discourses involved. Criminalising Discourse constructed FDV as historical actions or events, allowing FDV to be connected to the past rather than to the individual. Social Justice Discourse complimented this perspective by moving FDV into the public domain where it was able to escape specific social bounds or the mind and body of individuals.

**Table 4:** Object Oriented Narratives: Documents and discourses

Documents	Problem Discourses	Solution Discourses
NSW16 (State) [54]	**Social Justice** *Criminalising*	**Criminalising** *Social Justice*
NT20 (Clinical Practice) [56]	**Criminalising**	**Criminalising** **Social Justice**

Bold – dominant; *Italics* – non-dominant

While the two discourses are supportive in this way, they also act as counter discourses. Criminalising Discourse acknowledges the importance of the individual and emphasises punitive actions, making the need for individual accountability a priority. In contrast to this, Social Justice Discourse takes a generalising and forgiving stance which encourages expectations for individual change and recovery. The combination of these discourses can ‘unstick’ an individual from a ‘bad’ or ‘criminal’ position, while still allowing that person to be held accountable for a past action. Consequently, the narrative opens up the possibility for subject empowerment through expectations of change.

This type of narrative can provide a sense of balance, bringing to mind a dialectical approach of acceptance and change. Greater emphasis is placed on perpetrator accountability, while solutions are expected to lead every subject towards a positive future. Emphasis on the object rather than the subject may allow individuals to move between positions and avoid assumptions attached to rigid subject definitions. This may encourage health professionals to look past individual characteristics and focus more on the specific actions or outcomes involved within a single incident of FDV, potentially reducing the chances of stereotypical responses or problematic assumptions. Despite this, Criminalising Discourse allows for the object to be evidenced via the individual, potentially preventing health professionals from seeing beyond a single presentation and encouraging them to overlook long-term patterns or non-physical forms of abuse. This narrative may also encourage health professionals to act as heroes or detectives, disempowering subjects by over-riding their individual rights or preferences for the sake of minimising ‘risk’.

### Societal narratives

These narratives focused on the problem of inequality, making society the target problem and decreasing the visibility of individual subjects and health professionals. Table 5 below outlines the discourses involved in 2 documents which had a Societal Narrative. Social Justice Discourse provided a heavy and consistent influence as a dominant discourse within problem and solution streams of both documents. This emphasised the object of ‘inequality’ which was placed within a public domain, making everyone responsible for change. The historical and societal perspectives of Feminine Oppression, Evolutionary and Public Epidemic Discourses all supported this construction. The generalising nature of these discourses meant that individual subjects and professionals tended to become diluted within a societal pool, making their positions or unique characteristics seem irrelevant.

**Table 5:** Societal Narratives: Documents and discourses

Documents	Problem Discourses	Solution Discourses
NGO15 (National) [53]	**Social Justice** **Public Epidemic** *Evolutionary*	**Social Justice** **Feminine Oppression** *Public Epidemic* *Evolutionary*
WA20 (State) [57]	**Social Justice** **Evolutionary** *Public Epidemic* *Criminalising*	**Social Justice** **Evolutionary** *Public Epidemic* *Criminalising*

Bold – dominant; *Italics* – non-dominant

Criminalising Discourse provided a counter perspective within 1 document, highlighting the importance of individuals via perpetrator accountability and increasing the focus on FDV. The deficit tendency of this discourse seemed to be held back by other discourses however, with Evolutionary, Public Epidemic and Social Justice Discourses isolating the object from individuals and emphasising optimistic expectations for growth and recovery. This highlighted solutions and positive outcomes, moving the focus beyond the problem and making punitive actions seem like a positive step forward.

These narratives provide a feel-good impression of hope and certainty, where everyone is gathered together to follow a long journey of discovery that will lead to change. There is emphasis on leadership which appears to be Government or community-based, sidelining everyone else who is bulked within the subject of ‘society’. Individual subjects and health professionals therefore seem to be simultaneously responsible and disempowered as they appear to be grouped as social responders within society and FDV is moved out of private and health-related settings.

## Discussion

Foucauldian theories recognise the abstract, dynamic and interconnecting nature of discourses which can evolve, erode, combine and transform within a sociohistorical and institutionalised context [62]. The overlapping and conflicting nature of the discourses found within these policy documents highlights the potential for both shared discursive origins and the transformation of meanings due to different sociohistorical contexts. Earlier discourses which dominated Deficit Subject Narratives appeared to contain strong historical influences from empiricism, structuralism and biomedical epistemologies, with the tendency for discourses to focus on ‘factual’ evidence, categorical assumptions and generalising actions of control over individuals. The tendency to ‘govern’ or control individuals or groups continued to be a common feature over time, occurring within every narrative theme, with only the collective leadership position of Social Justice Discourse offering a glimpse of resistance through the recognition of marginalised communities as ‘leaders’. The colonising undertones of these narratives signifies potential negative implications for First Nations people of Australia whose values and strengths may be undermined by the authoritarian nature of Government institutional constructions. Despite the potential for similar sociohistorical influences, each discourse is distinguished by a unique object-subject-context construction, suggesting historical deviations and creating the potential for resistance in regards to dominant discursive meanings. This became more evident within later documents that appeared to be influenced by post-structural epistemologies, creating the fluidity in meaning that characterised Object Oriented and Societal Narratives.

While the critical nature of the discourse analysis revealed the possibilities for problematic assumptions, each discourse has the potential to produce a variety of outcomes, depending on the congruity between the assumptions generated by the discourse and the practical context in which the discourse is enacted. For example, Biomedical Deficit Discourse may be problematic when applied in situations where an individual has abilities and strengths; however, the discourse may be extremely valuable when an individual’s life is immediately threatened and interventions of control over the body are necessary for survival. A single discourse could therefore be simultaneously valuable and problematic when enacted ‘on the ground’ within a rural ED, highlighting the importance of considering situational, spatial and functional context when analysing discourses within clinical settings. The narrative analysis also demonstrated how the productive potential of each discourse could differ when combined with other discourses, highlighting the complexity of discourse within institutional policies and practices. These results stress the need for future research which provides a detailed examination of discursive constructions within rural ED environments and which acknowledges the importance of context when determining their productive potential.

### A historical transition in Australia’s understanding of FDV

A focused sociohistorical analysis of these discourses and narratives was done to examine how policy meanings may have changed between 2006 and 2020, and to explore possibilities for why certain meanings arose within particular periods of time, jurisdictions and policy levels. The historical analysis illustrated a period of substantial growth in Australia’s understanding of FDV, with potentially significant changes to assumptions of agency and value for both individual subjects and health professionals. As indicated in Table 2, earlier policy documents (i.e. 2006 – 2014) tended to combine Biomedical Deficit, Deficit Female and Criminalising Discourses, creating Deficit Subject Narratives that encouraged stereotypical and individualised deficit constructions of FDV. These narratives reflected concepts of deficit discourses which have been previously discussed within international and Australian literature related to particular groups, including patients [75], parents [105], women [96, 98] and First Nations people [18, 19]. Previous research investigating Australian policies and practices has revealed concerns related to deficit constructions, including the tendency for stereotypical concepts and responses which overlooked the importance of intersectionality and diversity [27]. From 2015 onwards, Object Oriented and Societal Narratives dominated, with Social Justice, Public Epidemic and Evolutionary discourses reducing the rigidity of meanings through community-based and contextualised perspectives of FDV. This allowed for greater variability in object meaning and the possibility of empowerment for individuals or groups.

The year 2015 may have marked a turning point where problematic discourses related to FDV were publicly challenged and possibilities for new meanings strongly emerged. NGO15 [53] provided a societal perspective of gender inequality which contrasted with the Deficit-Subject Narratives of preceding Government documents. Being the only document co-authored by non-Government bodies, NGO15 [53] appears to represent broader societal standpoints and a process of meaning making which may have reduced the influence of Government. The rigidity and consistency of earlier documents potentially illustrates Foucault’s notion of institutional power where internal processes maintain problematic assumptions when systems are not permeable to external discursive influences [8]. This can partly explain how some Government policies may not ‘match-up’ to the meanings and needs of individuals and groups within Australian society.

A review of literature and public media during this time suggests that the Me Too Movement may have influenced changes to discursive constructions of FDV within Australia. The Me Too Movement generated greater public awareness and rejection of sexual harassment and other forms of abuse (particularly for women), demonstrating how social discourses can dramatically alter meanings and actions for individuals and groups around the world [107]. An online article published by the National Sexual Assault Domestic Family Violence Counselling Service (referred to as ‘1800RESPECT’) in Australia suggested that the Me Too Movement contributed to a marked rise in the need for services between 2014 and 2018 [108], potentially demonstrating the movement’s impact on FDV subjects who were empowered to act differently. A research report by Australia’s National Research Organisation for Women’s Safety in 2016 provides an example of how problematic discourses within Australian society were being challenged at this time [109]. This report highlighted concerning portrayals of FDV within Australian media, with the results mimicking some of the deficit characteristics of Criminalising and Biomedical Deficit discourses, such as the individualising and decontextualizing of FDV, and assumptions which encouraged survivors to take responsibility for FDV while perpetrators remained invisible.

NT18 [55] may provide an example of how institutional meanings can stagnate within certain jurisdictions and despite broader societal movements. It stands out within Table 2 as a later document which maintained a Deficit Subject Narrative. This document may highlight the stubborn and powerful nature of institutional discourses which can resist external influences [8]; however, this document might also reflect the power of political events, as the document’s ‘child safety’ focus appears to challenge concerns outlined within a 2017 report that identified problematic responses to children within Northern Territory Government services [110]. Two years after NT18’s [55] publication, NT20’s [56] Object Oriented Narrative moves the Northern Territory beyond the deficit subject construction, suggesting that this jurisdiction may be opening up to alternative understandings of FDV or leaving behind the defensive position that appeared within the earlier document.

Broader social movements, political systems and events, and the advancement of knowledge via research may have therefore combined to alter the meanings of FDV within Australia. The introduction of new meanings might also signify enhanced input from traditionally marginalised groups, such as First Nations people who appear to have influenced the latest document included in the current study (WA20 [57]). Input from First Nations communities has particular relevance for addressing FDV within remote Australia, as they represent 18% and 47% of remote and very remote populations respectively [31]. First Nations communities are also more likely to be hospitalised for FDV compared to non-Indigenous Australians (32 times more likely for women and 23 times more likely for men) [111]. In more recent years, there appears to be increasing recognition of under-represented groups in discussions of FDV, including communities in rural and remote areas of Australia [30]. The 2016 International Conference on Practice and Policy in the Prevention of Violence against Women and their Children (hosted in Australia) highlighted the importance of recognising societal inequality and intersectionality, with suggestions that FDV-related solutions move away from a one-size-fits all approach and towards greater specificity for under-represented groups [112]. Four years later, the WA20 [57] document hints at this endeavour, being the only document within the current study to construct the collaborative leadership position of Social Justice Discourse which provided impressions of collaboration with First Nations people and created possibilities for multiple avenues of change. Theoretical influences from minority groups such as LGBTQIA + (e.g. Queer Theory) or First Nations people (e.g. Indigenous Theories) may have helped to steer away from dichotomous and hierarchical constructions of FDV such as those related to binary gender that appeared within Deficit Subject Narratives.

### Implications for Emergency Department staff, patients and rural communities

The current study demonstrated how policy narratives could uniquely position individual subjects when considering their agency and their relationship with health professionals. Survivors appeared to be gradually relieved of responsibility and blame as constructions of FDV left behind stereotypical deficit subject positions. Their image was strengthened as survivors had position versatility and the potential for innate value and ability. Perpetrators appeared to become increasingly visible and accountable, with their potential for independent change becoming more evident within later documents. These changes occurred as the object of FDV was extricated from the body and mind of individuals, and was placed within a non-clinical and sociohistorical setting, demonstrating the potential benefits of meaning transition from Deficit Subject to Object Oriented or Societal Narratives. New meanings appear to encourage acceptance of diversity, as FDV takes on a fluidity which may allow it to be adapted and moulded to suit a variety of people. Rather than being blamed and punished for their own suffering, rural communities could become experts in regards to themselves and what FDV means for them, potentially teaching health professionals the value of community understandings and actions which could inform clinical practice within an Emergency Department (ED). This creates the chance for greater specificity for Australian policy and clinical practice which has the potential to benefit rural communities.

To gain an understanding of how these meanings may apply to a rural ED setting, further jurisdictional level comparisons were done. As outlined in Table 2, none of the clinical practice policies had a Societal Narrative, with all 3 having either a Deficit Subject (NT20 [56]) or Object Oriented Narrative (NSW06 [50] and WA14 [52]). This highlighted the importance of purpose and context when considering the practical applications of policy narratives within clinical settings. In order to inform practice, health policies may need to be useful tools for health professionals. By elevating their position to ‘expert’, policies can empower and entice health professionals to comply with suggestions by convincing them that they will have the privileges, knowledge and skills needed to control the ‘problem of FDV’. Deficit Subject and Object Oriented Narratives both represent this purpose, increasing the agency of health professionals by giving them the potential to make decisions and take action on behalf of patients.

In contrast, Societal Narratives appear to position health professionals within a broad societal group, erasing their ‘expertise’ and making them seem redundant in constructions of FDV. They appear to be positioned on equal ground with other members of Australian society, with responsibility for action as citizens of their communities. The invisibility of health professionals could lead to their exclusion or minimisation within broader societal discussions of FDV, potentially maintaining problematic assumptions that have previously been reflected within ED practices [82]. Related funding and resources may also be redirected to ‘specialist services’, leaving rural ED’s without the necessary training or supports to respond effectively to FDV. Societal Narratives remove FDV from the clinical environment, making it difficult to comprehend how they could benefit clinical practice; except to suggest that health professionals play an equally important role in shaping local understandings of FDV. Taking this perspective, the institutional discourses and narratives that influence responses from health professionals within the context of a rural ED may be crucial when considering the social translation of meaning and agency for individuals within these marginalised areas. Societal Narratives may also open up the possibility for health professionals to learn from valuable discourses and narratives located within their rural communities. The potential benefits to individuals and communities notwithstanding, this could present challenges within public institutional settings which may struggle to support flexible and non-hierarchal systems and processes. Without the necessary resources and systems, typically understaffed rural hospitals may be pushed to take on more work by reaching out to their communities in an endeavour to build a shared understanding of FDV. The authoritarian nature of these narratives also raises scepticism regarding whether marginalised communities could influence institutional environments.

## Conclusion

The current study aimed to critically examine Australian Government family and domestic violence (FDV) policies to explore how they might impact on Emergency Department (ED) health professionals and individuals located within rural communities. The study highlights how institutional systems and broader societal movements might significantly influence understandings and responses to FDV when policy meanings are applied to a rural ED setting. There was considerable variability in terms of policy meaning, with each document being influenced by a unique combination of discourses which constructed Deficit Subject, Object Oriented or Societal Narratives. These narratives significantly changed the problem object and the position of individuals and health professionals.

Sociohistorical and political influences appeared to play an important role in shaping policy narratives. As the meaning of FDV changed over time, the target object was removed from the private clinical setting and placed within the public domain. The agency of health professionals declined as they transitioned from elite ‘clinical experts’ to ‘social responders’ alongside other societal members. Conversely, FDV subjects and rural communities were uplifted within the Object Oriented and Societal Narratives of later documents, leaving behind deficit assumptions and enabling individuals and communities to step into valued positions. While the application of Societal Narratives could pose challenges for health professionals in rural ED’s who may not be sufficiently supported to alter institutionalised responses to FDV, the increasing potential for community engagement provides some hope that policy-to-practice disconnections could be overcome. As Government systems open up to outsider influences, policies can gain greater specificity and health professionals could be encouraged to learn from community members; however, if ED resources are not consistent with this endeavour, such claims may be considered as rhetoric. Further research examining the effectiveness and translation of FDV policies ‘on the ground’ in rural communities would aid in understanding the productive potential of these narratives and discourses.

## Data Availability

The policy document data that support the findings of this study are available from the following corresponding sources: **Table:** 

Data Reference	Source
COAG11 [2]	Council of Australian Governments (COAG). (2011). *National plan to reduce violence against women and their children 2010–2022*. Canberra ACT: Commonwealth of Australia Retrieved from https://www.dss.gov.au/women/programs-services/reducing-violence/the-national-plan-to-reduce-violence-against-women-and-their-children-2010-2022.
WA20 [57]	Department of Communities. (2020). *Path to safety: Western Australia's strategy to reduce family and domestic violence 2020–2030*. Perth: Government of Western Australia Retrieved from https://www.communities.wa.gov.au/media/2766/fdv-strategy.pdf.
NSW12 [51]	New South Wales Government. (2012). *It stops here. Standing together to end domestic and family violence in NSW: The NSW Government's domestic and family violence framework for reform*. NSW Government Retrieved from https://www.women.nsw.gov.au/__data/assets/file/0003/289461/It_stops_Here_final_Feb2014.pdf.
NSW06 [50]	New South Wales Health. (2006). *Domestic violence: Identifying and responding*. Sydney NSW: New South Wales Government Retrieved from https://www1.health.nsw.gov.au/pds/ActivePDSDocuments/PD2006_084.pdf.
NT18 [55]	Northern Territory Government. (2018). *Domestic, family and sexual violence reduction framework 2018–2028*. Casuarina: Northern Territory Government Retrieved from https://territoryfamilies.nt.gov.au/__data/assets/pdf_file/0006/464775/Domestic,-Family-and-Sexual-Violence-Reduction-Framework.pdf.
NT20 [56]	Northern Territory Government. (2020a). *Northern Territory domestic and family violence risk assessment and management framework*. Darwin NT: Northern Territory Government Retrieved from https://tfhc.nt.gov.au/domestic,-family-and-sexual-violence-reduction/ramf.
NSW16 [54]	NSW Ministry of Health. (2016). *New South Wales domestic and family violence blueprint for reform 2016–2021: Safer lives for women, men and children*. North Sydney: New South Wales Ministry for Health Retrieved from https://www.women.nsw.gov.au/strategies/nsw-domestic-and-family-violence/domestic-and-family-violence-blueprint.
NGO15 [53]	Our Watch, Australia's National Research Organisation for Women's Safety (ANROWS), & VicHealth. (2015). *Change the story: A shared framework for the primary prevention of violence against women and their children in Australia* Melbourne, Australia: Our Watch Retrieved from https://d2bb010tdzqaq7.cloudfront.net/wp-content/uploads/sites/2/2019/05/21025429/Change-the-story-framework-prevent-violence-women-children-AA-new.pdf. The following policy document data that support the findings of this study is no longer publicly available. This document can be accessed by contacting the corresponding author on request:

**Table:** 

Data Reference	Source
WA14 [52]	Department of Health Western Australia. (2014). *Guideline for responding to family and domestic violence*. Perth: Department of Health Western Australia. https://ww2.health.wa.gov.au/About-us/Contact-us.

## Abbreviations

AIHW: Australian Institute of Health and Welfare
CDC: Centres for Disease Control and Prevention
COAG: Coalition of Australian Governments
COAG11: Policy document used in analysis
ED: Emergency Department
FDV: Family and domestic violence
NGO: Non-government organisations. Organisations which are independent of Government bodies
NGO15: Policy document used in analysis
NSW: New South Wales (state within Australia)
NSW06: Policy document used in analysis
NSW12: Policy document used in analysis
NSW16: Policy document used in analysis
NT: Northern Territory (territory within Australia)
NT18: Policy document used in analysis
NT20: Policy document used in analysis
VIC: Victoria (state within Australia)
WA: Western Australia (state within Australia)
WA14: Policy document used in analysis
WA20: Policy document used in analysis
